# Urinary Eosinophil Protein X in Childhood Asthma: Relation with Changes in Disease Control and Eosinophilic Airway Inflammation

**DOI:** 10.1155/2013/532619

**Published:** 2013-01-15

**Authors:** Marianne Nuijsink, Wim C. J. Hop, Peter J. Sterk, Eric J. Duiverman, Johan C. De Jongste

**Affiliations:** ^1^Department of Pediatric Respiratory Medicine, Juliana Children's Hospital, HAGA Teaching Hospital, P.O. Box 60605, 2506 LP, The Hague, The Netherlands; ^2^Department of Biostatistics, Erasmus University Medical Centre, P.O. Box 2040, 3000 CA, Rotterdam, The Netherlands; ^3^Department of Respiratory Medicine, Academic Medical University of Amsterdam, P.O. Box 22660, 1100 DD, Amsterdam, The Netherlands; ^4^Department of Pediatric Respiratory Medicine, Beatrix Children's Hospital, University Medical Centre Groningen, P.O. Box 30001, 9700 RB, Groningen, The Netherlands; ^5^Department of Pediatric Respiratory Medicine, Sophia Children's Hospital, Erasmus University Medical Centre, P.O. Box 2060, 3000 CB, Rotterdam, The Netherlands

## Abstract

The aim of this study was to assess cross-sectional and longitudinal correlations between uEPX and other markers of asthma control and eosinophilic airway inflammation. *Methods*. We measured uEPX at baseline, after 1 year and after 2 years in 205 atopic asthmatic children using inhaled fluticasone. At the same time points, we assessed symptom scores (2 weeks diary card), lung function (forced expiratory volume in one second (FEV_1_)), airway hyperresponsiveness (AHR), and percentage eosinophils in induced sputum (% eos). *Results*. We found negative correlations between uEPX and FEV_1_ at baseline (*r* = −0.18, *P* = 0.01), after 1 year (*r* = −0.25, *P* < 0.01) and after 2 years (*r* = −0.21, *P* = 0.02). Within-patient changes of uEPX showed a negative association with FEV_1_ changes (at 1 year: *r* = −0.24, *P* = 0.01; at 2 years: *r* = −0.21, *P* = 0.03). Within-patient changes from baseline of uEPX correlated with changes in % eos. No relations were found between uEPX and symptoms. *Conclusion*. In this population of children with atopic asthma, uEPX correlated with FEV_1_ and % eos, and within-subjects changes in uEPX correlated with changes in FEV_1_ and % eos. As the associations were weak and the scatter of uEPX wide, it seems unlikely that uEPX will be useful as a biomarker for monitoring asthma control in the individual child.

## 1. Introduction

Eosinophilic protein X is produced by activated eosinophils and can be measured in blood, stools, and urine (uEPX) [1, 2]. Elevated uEPX has been reported in asthmatics with or without allergy [3, 4], with great overlap between healthy controls and asthmatics [5, 6]. A relation with asthma symptoms and lung function has been shown, and uEPX increases significantly during asthma exacerbations [7–9]. A reduction in uEPX has been reported after starting inhaled corticosteroids [10]. Therefore, it has been suggested that measuring urinary EPX may be useful for monitoring the effect of ICS in asthma [3, 10, 11]. 

Since measuring uEPX is completely noninvasive and independent of patient's cooperation, it has the potential to be a feasible tool in asthma management in young children. Several studies have demonstrated cross-sectional correlations between uEPX and conventional clinical markers of asthma control and airway inflammation [3, 4, 12]. Previously, we reported these cross-sectional data measured at baseline in part of the present study population [13]. Data on longitudinal changes in uEPX within subjects compared to changes in markers of asthma control or airway inflammation were examined in only one study in 14 children [3].

We analysed cross-sectional and longitudinal uEPX data from a long-term follow-up study in children with atopic asthma [14]. We hypothesised that changes in uEPX would be related to changes eosinophilic airway inflammation and could provide information that is additional to symptoms and lung function.

## 2. Methods

### 2.1. Subjects

Asthmatic children took part in a randomised controlled multicentre trial (CATO: Children Asthma Therapy Optimal) reported in detail previously [14]. Briefly, 288 children with a documented clinical history of moderately severe asthma were recruited from 15 paediatric clinics including 7 university hospitals in the Netherlands. Al patients had a positive, class 1, or more radioallergosorbent test result for one or more airborne allergens.

During a run-in phase, all patients were treated with 100 or 250 *μ*g fluticasone bid depending on their equivalent treatment before the study. Children were selected on the basis of symptoms and/or AHR, and 210 children were randomised to a reference strategy (adjustment of treatment on symptom score) or an AHR strategy (treatment adjusted on the basis of AHR and symptom score). During the 2-year follow-up study, 3 monthly visits were performed. At each visit symptoms scores (diary card over the last 2 weeks), forced expiratory volume in one second (FEV_1_), and methacholine challenge results were obtained, and medication (five levels: fluticasone 100 or 200 *μ*g per day or fluticasone/salmeterol 200/100, 500/100, or 1000/100 *μ*g per day (Flixotide Diskus or Seretide Diskus, GlaxoSmithKline, UK) adjusted according to the algorithms of the reference- and AHR strategy.

For the present study, “baseline” is defined as the moment of randomisation for the CATO study.

### 2.2. Symptom Scores

 Patients filled in a diary card daily during 2 weeks before each 3-month visit. Cough, shortness of breath, and wheezing during night and day were recorded on a 4 point scale (0 = no symptoms to 3 = severe symptoms interfering with activity or sleep) [15]. The percentage of symptom-free days defined as score 0 for cough, wheeze, and shortness of breath was calculated.

### 2.3. AHR and Spirometry

were measured during each clinic visit [16]. Study medication was stopped 36 hours before lung function and AHR testing. AHR was tested by methacholine challenge using a dosimeter method [17]. The provocation dose causing a 20% fall in FEV_1_ from baseline (PD_20_) was calculated from a log dose-response plot by linear interpolation. 

### 2.4. Sputum Induction and Processing

 Sputum induction was attempted in 8 of the 15 participating centres. Sputum was induced according to a standardised method by inhaling an aerosol of hypertonic sodium chloride 4.5% w/v [18, 19]. Differential cell counts of cytospins were performed and samples containing more than 80% squamous cells were excluded from the analysis [18].

### 2.5. Urinary Eosinophil Protein X

Spot samples of urine were collected at randomisation (= baseline), after a treatment period of 1 year, and after 2 years (the end of the study), and immediately stored at −20°C. EPX was determined using a commercial enzyme-linked immunosorbent assay (ELISA) for human EPX in 50-fold diluted samples (Medical and Biological Laboratories, Nakaku Nagoya, Japan). The essay's sensitivity was 0.62 ng/mL. Urinary creatinine levels were measured by the alkaline picrate method (Roche, Mannheim, Germany). Urinary EPX concentrations were expressed as *μ*g per mmol creatinine.

### 2.6. Statistical Analysis

Repeated measurements Anova was used to evaluate associations between the various markers and uEPX levels. The same method was used to assess changes from baseline. With this method (SAS PROC MIXED) the information about the associations at the different study time points can be combined and the method allows for uEPX not being available at all three time points for each patient. In these analyses, uEPX values, % eosinophils in sputum, and fluticasone doses were transformed logarithmically in order to get approximate normal distributions and to reduce the effect of outlying observations, and consequently results for these parameters are expressed as geometric means. Percentage eosinophil values equal to 0 were replaced by 0.1% in order to allow the logarithmic transformation. In a substantial part of the study population, especially at time points T1 and T2, PD_20_ was higher than the highest dose methacholine used (1570 *μ*g). For that reason PD_20_ data were arbitrarily dichotomized in non-AHR (PD_20_ > 300 *μ*g) and AHR (PD_20_ < 300 *μ*g). Correlation coefficients calculated at the different time points are Spearman's rank correlations. *P* = 0.05 (two-sided) was considered the limit of significance.

## 3. Results

**Figure 1:**
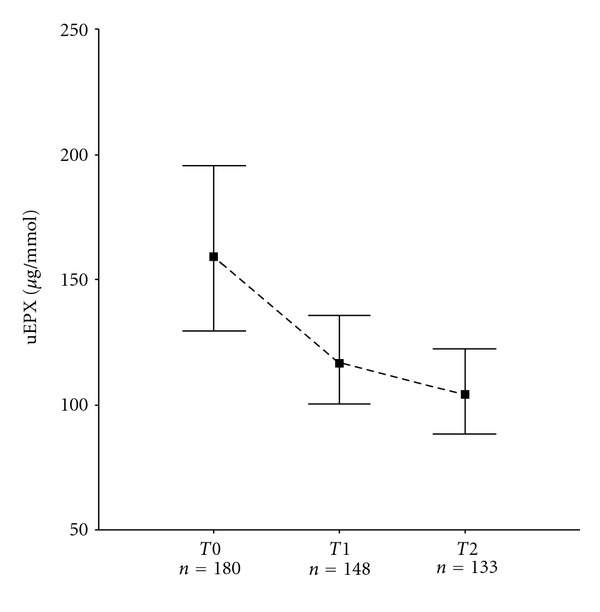
uEPX levels. Geometric mean uEPX (per mmol creatinine) levels with 95 percent confidence intervals by measurement visit (Anova estimates). T0: baseline; T1: after a treatment period of 1 year; T2: after a treatment period of 2 years. T1 versus T0: *P* = 0.01; T2 versus T1: *P* = 0.18; T2 versus T0: *P* < 0.001.

Urinary EPX was measured at least once in 205 children. Patient characteristics at baseline are given in Table 1. In total 461, uEPX samples were analysed during the study. During the 2-year treatment period, the geometric mean uEPX significantly decreased from 159 *μ*g/mmol to 104 *μ*g/mmol (*P* < 0.001) (Figure 1). Anova showed a significant treatment effect after 1 year: in the AHR strategy group, uEPX had decreased significantly more than in the reference strategy group, with a 29% lower geometric mean EPX level at that time point (*P* = 0.03). No treatment effect was found at year 2 (*P* = 0.26). Further analysis showed that at 1 year, there was a significant fluticasone dose effect, and this effect was not found at baseline or at year 2. At 1 year a twofold higher dose was associated with a 25% lower uEPX level (95% CI: 15%–34%; *P* < 0.001). Children treated according to the AHR strategy had received significantly higher doses of fluticasone at 1 year (geometric means 580 versus 412 ug/day; Mann-Whitney test, *P* = 0.01). After adjustment for the higher dose using Anova, no significant treatment effect remained.

### 3.1. Urinary EPX versus Parameters of Asthma Control (Tables 2 and 3)

**Figure 2:**
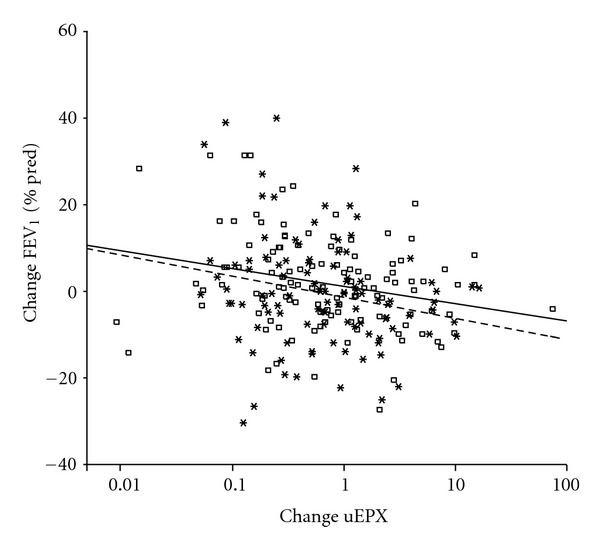
Scatterplot of within-patient changes from baseline in forced expiratory volume in 1 second versus changes in uEPX. Squares and the solid regression line pertain to changes between baseline and 1-year-followup; asterisks and the dashed line represent changes between baseline and 2 years. Changes of uEPX are shown as ratios; changes of FEV_1_ are shown as absolute differences of percentage predicted values.

 No correlations were found between uEPX and the percentage symptom-free days or changes in uEPX and changes in percentage symptom-free days. Anova also did not show significant associations. Cross-sectional analysis showed weak negative correlations of the levels of uEPX and FEV_1_ (baseline: *r* = − 0.18, *P* = 0.02; at 1 year: *r* = − 0.25, *P* = 0.002; at 2 years: *r* = − 0.21, *P* = 0.016, resp.). Anova showed that the significant associations did not differ between the three time points and adjusting for ICS dose did not change this. Also the changes from baseline of uEPX and FEV_1_ were significantly related with each other (at 1 year *r* = −0.24, *P* = 0.01 and at 2 years; *r* = −0.21, *P* = 0.03) (Figure 2). As compared to baseline, a tenfold increase in uEPX was associated with a decrease of FEV_1_ (% pred) of 2.8 percentage points (95% CI : 0.3 to 5.3; *P* = 0.026) and this decrease did not significantly differ between the two time points (*P* = 0.65). A similar result was found when changes from the previous assessment were evaluated.

**Figure 3:**
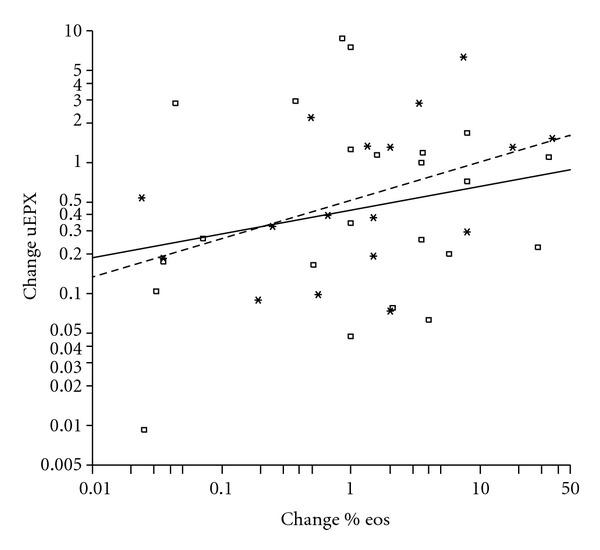
Scatterplot of within-patient changes from baseline in uEPX versus changes in percentage sputum eosinophils. Squares and the solid regression line pertain to the changes between baseline and 1 year (*n* = 23); asterisks and the dashed line represent changes between baseline and 2 years (*n* = 17). Changes of uEPX and percentage sputum eosinophils are both shown as ratios.

 At all visits, uEPX levels were higher in patients with AHR (PD_20_ methacholine < 300 *μ*g methacholine) than in patients without AHR. At 1 year this difference was statistically significant, with 13% higher uEPX in patients with AHR (*P* = 0.006) (*P* = 0.08, *P* = 0.10, resp., at baseline and after 2 years treatment). This effect remained after adjustment for dose. With regard to percentage eosinophils in sputum a significant correlation with uEPX was found at 2 years (*r* = 0.51, *P* < 0.001, *n* = 33). Anova showed a significant overall association between uEPX and percentage sputum eosinophils. A twofold higher value of sputum eosinophils was associated with a 14% increased value of uEPX (95% CI: 6%–22%; *P* < 0.001), and this relation did not significantly differ between the 3 time points (*P* = 0.11), nor was the relation affected by ICS dose. Although changes from baseline of uEPX and percentage sputum eosinophils did not significantly correlate with each other at the separate time points, a significant relation was found combining both time points using Anova. A within-patient doubling of sputum eosinophils was associated with a mean increase of uEPX of 16% (95% CI: 0.1%–36%; *P* = 0.049), and the ICS dose did not affect this relation. The individual variations however are large (Figure 3).

## 4. Discussion

We prospectively assessed changes in uEPX in relation to changes in asthma control within subjects over an observation period of 2 years. We found in atopic asthmatic children that changes in uEPX measured after 1 and 2 years related significantly with changes in FEV_1_ and % sputum eosinophils, but not with changes in symptoms. Changes in uEPX correlated with changes in ICS dose. 

Conflicting data have been reported about cross-sectional relationships between uEPX and FEV_1_. Previously we described the results of cross-sectional analysis at baseline in the present study population [13]. We found a weak inverse correlation between uEPX and FEV_1_. With the present study, we confirmed the relation between uEPX and FEV_1_ at different time points. Moreover, we showed that changes in uEPX and changes FEV_1_ were related. This is in line with the significant correlation between these parameters found by Lugosi et al. in 14 asthmatic children [3]. However the interval of their measurements was much shorter (1-2 months). It has been shown that correlations between eosinophilic airway inflammation and lung function are weak and may only reach significance in large populations [4, 6, 20, 21]. Clearly FEV_1_ is dependent on many more factors than eosinophilic inflammation alone.

The correlation between uEPX and sputum eosinophils was weak, and this is in line with the relations between sputum ECP, sputum eosinophils, and uEPX described by Mattes et al. [12]. Weak correlations seem to be the rule when different markers of eosinophilic inflammation are compared [22]. Moreover, uEPX is secreted by activated eosinophils, and it is therefore possible that uEPX provides information about eosinophil activation rather than numbers.

We found no relation between uEPX and symptoms. A significant relation between uEPX and symptoms has been described during acute asthma exacerbations, spontaneously or provoked by ICS withdrawal [23]. Also, significantly higher uEPX levels were found in symptomatic asthmatic children than in asymptomatic children [1, 3, 4]. However, a 6-month follow-up study in 14 children with mild asthma found, no significant association was found [9]. So the severity of symptoms should be taken into account when correlating uEPX and symptoms. In our study population, the symptom scores were low and this may explain the apparent discrepancy with these previous studies. It could well be that relations between uEPX and parameters of asthma control could have been influenced by other factors as well, including seasonal variations, severity of allergy, and asthma and atopic dermatitis [24, 25]. The use of varying doses of ICS is another possibility, for which we have corrected. 

What are the clinical implications of our findings? As uEPX does not correlate with symptoms, but does correlate with sputum eosinophils, we could argue that uEPX might provide additional information on activity of eosinophilic airway inflammation. However, the correlations were weak and the scatter of individual uEPX values wide. Therefore, it seems unlikely that uEPX will be useful as a biomarker for monitoring asthma in the individual child.

## Figures and Tables

**Table 1:** Characteristics of the 205 children at baseline.

Age (y)	10.4 [6.4–16.8]
Gender (m/f)	118/87
Daily dose fluticasone 200 *μ*g/500 *μ*g	116/89
uEPX (*μ*g/mmol creatinine) (*n* = 180)	184 (2–3114)
Symptom-free days (%)	50 (0–100)
FEV_1 _(% pred)	97 (56–136)
PD_20_ methacholine (*μ*g)	72 (1–>1570.0^#^)
Sputum eosinophils (%) (*n* = 49)^†^	1 (0–72)

Values are median (range).

FEV_1_: forced expiratory volume in 1 second; PD_20_ methacholine: provocative dose of methacholine causing FEV_1 _fall 20% from baseline.

^#^12 children had not reached the 20% fall at the highest dose of 1570 *μ*g.

^†^79 patients had a total of 127% eos measurements combined with an uEPX measurement, and in 49 of these a baseline measurement was available.

Baseline characteristics form part of this group have been published previously in a cross-sectional study by Nuijsink et al. [13].

**Table 2:** Correlations between uEPX and clinical markers of asthma.

		*r*	*P* value
	T0	−0.04	0.96
Symptom-free days (%)	T1	0.03	0.68
	T2	0.09	0.34

	T0	−0.18	0.01
FEV_1 _(% pred)	T1	−0.25	<0.01
	T2	−0.22	0.02

	T0	0.19	0.19
Sputum eosinophils (%)	T1	0.19	0.20
	T2	0.51	<0.01

	T0	−0.14	0.08
PD_20_ methacholine (*μ*g)	T1	−0.20	0.02
	T2	−0.14	0.14

T0: at baseline; T1: after a treatment period of 1 year; T2: after a treatment period of 2 years; FEV_1_: forced expiratory volume in 1 second. Numbers evaluated for sputum eosinophils at T0, T1, and T2 are 49, 45, and 33, respectively. Data shown represent Spearman rank correlation coefficients.

Correlations at baseline (T0) in part of this group have been published previously in a cross-sectional study (Nuijsink et al. [13]).

**Table 3:** Correlations between the changes from baseline in uEPX and changes in clinical markers of asthma control.

		*r*	*P* value
Symptom-free days (%)	T1	−0.06	0.53
	T2	0.10	0.32
FEV_1_ (% pred)	T1	−0.24	0.01
	T2	−0.21	0.03
Sputum eosinophils (%)	T1	0.10	0.64
	T2	0.40	0.11

T0: at baseline; T1: after a treatment period of 1 year; T2: after a treatment period of 2 years. FEV_1_: forced expiratory volume in 1 second. The individual changes from baseline of uEPX and % eos are expressed as ratios. For FEV_1_ and symptom-free days, the changes are expressed as the absolute differences of the measured percentages. Numbers evaluable for the change from baseline of % eos at T1 and T2 are 23 and 17, respectively. Data shown are Spearman rank correlations.
